# Neutrophil-Derived IL-6 Potentially Drives Ferroptosis Resistance in B Cells in Lupus Kidney

**DOI:** 10.1155/2023/9810733

**Published:** 2023-05-27

**Authors:** Zechen Wang, Jiajia Shen, Kun Ye, Jingjie Zhao, Shaoang Huang, Siyuan He, Yujuan Qin, Lingzhang Meng, Jie Wang, Jian Song

**Affiliations:** ^1^Center for Systemic Inflammation Research (CSIR), School of Preclinical Medicine, Youjiang Medical University for Nationalities, Baise, Guangxi Province, China; ^2^Department of Renal Diseases, The People's Hospital of Guangxi Zhuang Autonomous Region, Nanning Guangxi Province, China; ^3^Life Science and Clinical Research Center, The Affiliated Hospital of Youjiang Medical University for Nationalities, Baise, Guangxi Province, China; ^4^Institute of Cardiovascular Sciences, Guangxi Academy of Medical Sciences, Nanning, Guangxi Province, China; ^5^Department of Renal Diseases, The Affiliated Hospital of Youjiang Medical University for Nationalities, Baise, Guangxi Province, China

## Abstract

Ferroptosis resistance is vital for B cell development, especially in inflammatory diseases, yet the underlying mechanism is still unclear. In this study, based on the scRNA-seq technique and flow cytometry, we discovered a proportion of neutrophils exhibited upregulated expression of the IL-6 and correlated with the expression of IL-6 receptor and SLC7A11 from B cells in lupus kidney. Moreover, we identified that in lupus kidney, neutrophils could provide IL-6 to facilitate ferroptosis resistance in B cells via SLC7A11, and inhibition of SLC7A11 could significantly enhance ferroptosis in B cells and could decrease B cell proliferation. This study helps understand the crosstalk between neutrophils and B cells in the kidney in the development of lupus.

## 1. Introduction

Ferroptosis is an iron-dependent form of regulated cell death characterized by the accumulation of lipid peroxides. Ferroptosis resistance, the ability of cells or tissues to evade ferroptotic cell death, has been implicated in the development of cell death and various diseases [1–4], yet its role in lupus still needs to be depicted. Lupus is an autoimmune disease characterized by hypergammaglobulinemia derived from pathogenic B cell behavior [5–7]. Illustrating the molecular mechanism of abnormal B cell, for example, the manner of B cell death, would help understand its role in driving the development of lupus.

It is known that B cell behavior is tightly regulated by the microenvironment [8, 9], for example, neutrophils [10, 11]. For example, in the spleen, neutrophil enters germinal center and provide BAFF, APRIL, IL-21, and PTX3 to stimulate immunoglobulin diversification and production [10, 12]. In the lymph node, neutrophils appeared in B cell zone and potentially help activate, and prolong the life time of B cells by providing BAFF and APRIL [13]. However, whether neutrophil modulate B cell behavior and the potential molecular mechanism is still less discussed.

In this study, we demonstrated that a population of neutrophils helps B cells suppress ferroptosis via SLC7A11 in the lupus kidney. Strikingly, blocking IL-6 significantly promoted ferroptosis of B cells and showed its therapeutic effect. Precisely modulating IL-6-expressing neutrophils in the kidney would be a potential therapeutic strategy to improve clinical treatment for lupus.

## 2. Materials and Methods

### 2.1. scRNA-Seq Analysis

The scRNA-seq data of murine glomeruli including those suffering from glomerular nephritis (GN) was retrieved from the NCBI GEO database under the accession ID GSE146912 [14]. The R package Seurat (v4.3.0) was used to process scRNA-seq data, and cells were clustered at resolution 0.6 after removing those genes expressed by less than 3 cells and those cells with less than 200 genes sequenced; min.pct = 0.25 and logfc.threshold = 0.25 were used to identify enriched genes that were expressed by each cell types. The R package CellChat (v1.5.0) was used to analyze inferred cell-cell communications.

### 2.2. Mice

C57BL/6 mice were purchased from the Changsha Tianqin Ltd. All experimental mice were bred under SPF conditions and 12-hour day/12-hour night cycle. Only 10-week-old male mice were used in this study. For induction of systemic lupus erythematosus, mice were introperitoneally injected with 500 *μ*l of pristane (Sigma-Aldrich, #P2870) [15]. For the control group, mice were injected with an equal volume of PBS. All the procedures used in this study were approved by the Ethical Committee of Youjiang Medical University for Nationalities.

### 2.3. Cell Culture

For cell culture, B cells and neutrophils were purified from kidneys with MACS cell separation beads (Myiltenyi, #130-090-862 and #130-097-658). Isolated B cells and neutrophil reached purity above 98% were used for co\culture at a ratio of 10 : 1 (B cells: neutrophils). Cells were placed in 96-well transwell plates (Corning, #CLS3388), in which B cells were placed at the bottom and neutrophils were placed on top and cultured in RPMI1640 (Sigma-Aldrich, #R8758) for 1 day. For culturing B cells with IL-6 (Cell Sgingaling, # 5216SC), a concentration of 10 ng/mL was used; for ferrostatin 1 (R&D, 5180) treatment, a concentration of 30 nM was used. The medium was supplemented with 10% of FBS (Sigma-Aldrich, #12106C) and 1% penicilin (Sigma-Aldrich, #V900929).

### 2.4. Flow Cytometry

For preparation of a single cell suspension, freshly isolated kidney tissues were cut into small pieces, digested with Collagen IV (25 mg/mL, Gibco, #1704) at 37°C for 45 minutes, and filtered through a 100 *μ*m stainless cell strainer. After blocking unspecific binding, samples were incubated with fluorescent-coupled antibodies. For intracellular staining, cells were fixed with 2% PFA for 15 minutes at room temperature, then permeabilized with permeabilization buffer (Invitrogen, #00-8333-56) at 4°C for 10 minutes, and incubated with fluorescent coupled antibodies. After washing twice with PBS/0.5%BSA, cells were resuspended in PBS and measured on the flow cytometer (Thermo Fisher Attune NxT).

### 2.5. Fluorescent Antibodies/Materials

The fluorescent coupled antibodies used in this study included CD11b-Pacific Blue (Biolegend, #101224), Ly6C-PE (Invitrogen, #12-5932-82), Ly6G-PE/Cy7 (Biolegend, #127618), Aldh2-FITC (Novus, #NBP2-70151F), Socs3-APC (Biorbyt, # orb1000608), fixable viability dye eFluor 660 (Invitrogen, #65-0864-14), FerroOrange (Dojindo, #F374), CFSE (Biolegend, #423801), IL6-PE (Biolegend, # 504503), B220-FITC (Biolegend, # 103205), Ki67-APC (Biolegend, # 350514), SLC7A11 (Invitrogen, #MA5-44922, inhouse coupled to A647), DAPI (Invitrogen, #D1306), Lipid Peroxidation/LiperFluo kit (Invitrogen, #C10445), and FerroOrange Kit (Amerigo, #F374).

### 2.6. Immunofluorescent Imaging

After removing the supernatant from cell culture, cells were fixed with 2% of paraformaldehyde (Sigma-Aldrich, #30525-89-4) at room temperature for 10 minutes, then cells were counterstained with DAPI (Invitrogen, #D1306), Lipid Peroxidation/LiperFluo kit or FerroOrange Kit according to the manufacturer's instructions. Immunofluorescent images were captured by the fluorescence microscope (Leica DMI300B). The fluorescent intensity was acquired by the software ImageJ.

### 2.7. qPCR

RNA was abstracted from cultured B cells with a commercial kit (TaKaRa, #9109) and performed by RT-PCR (TaKaRa, #RR047A) and qPCR (QUIGEN, #204056). The primers for SLC7A11 were as follows: SLC7A1-F: 5′-GGCACCGTCATCGG.

ATCAG-3′, SLC7A1-R: 5′-CTCCACAGGCAGACCAGAAAA-3′; the primers for GAPDH were as follows: GAPDH-F: 5′-AGGTCGGTGTGAACGGATTTG-3′, GAPDH-R: 5′-TGTAGACCATGTAGTTGAGGTCA-3′. The primers were purchased from Sangon Biotech Ltd. (Shanghai, China).

### 2.8. Quantification of GSH and GSSG

Intracellular glutathione (GSH) and oxidized glutathione (GSSG) were quantitatively measured by a commercial kit (DOJINDO Laboratory, #G263). Briefly, B cells were harvested after culture and spun down at 4°C and 300 g for 10 min. After washing twice with cold PBS, 80 *μ*l of 10 mM HCl solution was added to obtain lysate, then 20 *μ*l of 5% SSA was added and spun down at 4°C, 8000 g for 10 min. The lysate was diluted in 0.5% SSA. GSH and GSSG were measured according to the manufacturer's instruction [16].

### 2.9. Statistical Analysis

All the data were statistically analyzed by Graphpad Prism 6.0. The nonparametric test was used to analyze the differences among groups. Data were presented as mean ± SD. *p* < 0.05 was considered statistically significant.

## 3. Results

### 3.1. Neutrophil Heterogeneity in Lupus Kidney

**Figure 1:**
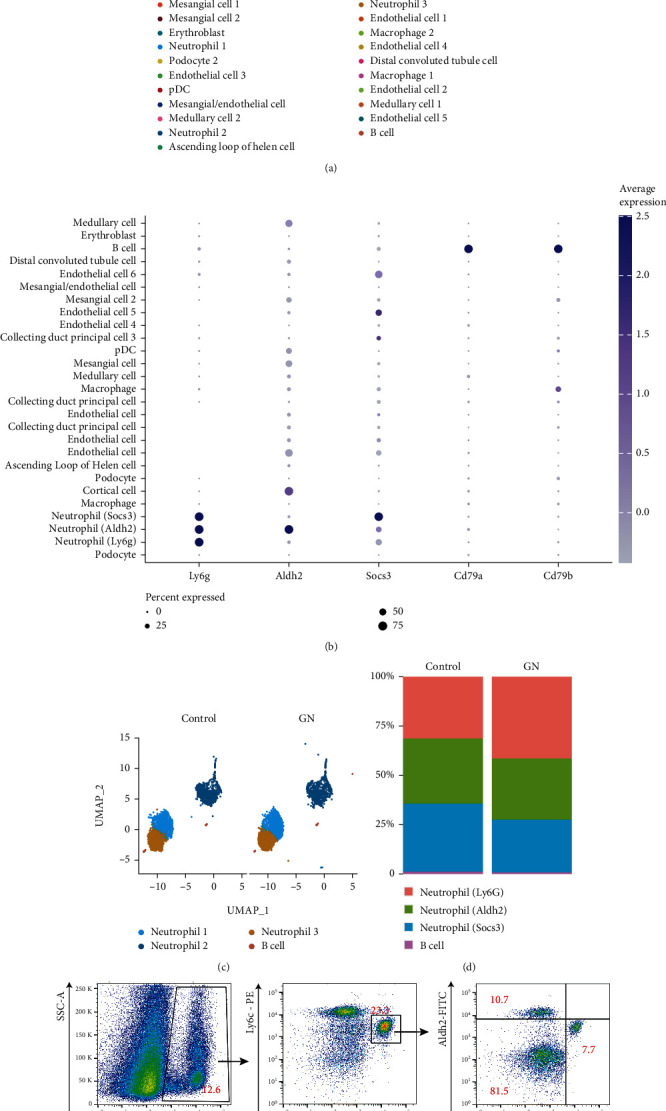
Identification of 3 neutrophil subclusters from lupus kidney. (a) UMAP plot showed 27 cell types in both control and lupus kidney. (b) 3 neutrophil subclusters and B cells were isolated from the whole kidney. (c) Dot plot showed the expression level of features for neutrophils and B cells. Cd79a and Cd79b for B cells, Ly6g for all neutrophils, and Aldh2 and Socs3 for newly identified 2 neutrophil subclusters. (d) Stacked bar plot showed the comparison of the frequencies of 3 neutrophil subclusters and B cells. (e) Flow cytometry analysis validated 3 neutrophil subclusters in kidney. Dead cells were removed by counterstaining fixable viability dye. (f) Dot plots showed the comparison of 3 neutrophil subclusters between control (*n* = 8) and lupus (*n* = 8) kidneys. Data represented similar results from at 3 independent experiments. ^∗∗∗^*p* < 0.001; ^∗∗^*p* < 0.01; ns: no significance.

GN usually occurred in lupus. To explore the heterogeneity of neutrophils in glomeruli in GN kidney, we reanalyzed the scRNA-seq data [14]. After vigorous quality control (Supplementary Figure 1A, 1B, 1C and 1D), 6043 cells from healthy controls and 8181 cell from GN kidney were integrated for downstreaming analysis. In total, 27 cell clusters were identified based on enriched genes expressed by each cell type (Figure 1(a), Supplementary table), including 3 neutrophil subpopulations and B cells. All the neutrophils were featured by high expression level of Ly6g; besides, two of them could be distinguished by high expression of Aldh2 and Socs3, respectively (Figure 1(b)). B cells were identified by high expression of Cd79a and Cd79b (Figure 1(b)). Global analysis of cell-cell communication among these 27 cell types showed increased interaction numbers but decreased interaction strength in GN, compared with controls (Supplementary figure 2A and 2B). To better explore the cell-cell communications between neutrophils and B cells in glomeruli suffering from GN, 3 neutrophil subpopulations and B cells identified from scRNA-seq data were isolated for downstreaming analysis (Figure 1(c)). Further comparison showed that the frequency of subcluster neutrophil (Ly6g) is much higher in lupus kidney (Figure 1(d)), indicating this subpopulation plays an active role in modulating the microenvironment of GN. It is known that GN is also a pathological feature occurred to lupus. To validate and to check whether these 3 neutrophil subpopulations exist in lupus, freshly isolated lupus kidney biopsies were analyzed by flow cytometry, and these 3 neutrophil subclusters were identified (Figure 1(e)). Based on flow cytometry analysis, we found the subcluster neutrophil (Ly6g) in the lupus kidney increased by almost 30%, compared with healthy control kidney, the neutrophil (Aldh2) increased by 8%, and the number of neutrophil (Socs3) was similar to healthy ones (Figure 1(f)). This suggested that neutrophil (Ly6g) could be the “main player” in the development of GN, comparing with the other two neutrophil subpopulations.

### 3.2. Neutrophil-Derived IL-6 Conducted a Major Signal between Neutrophils and B Cells in Lupus Kidney

**Figure 2:**
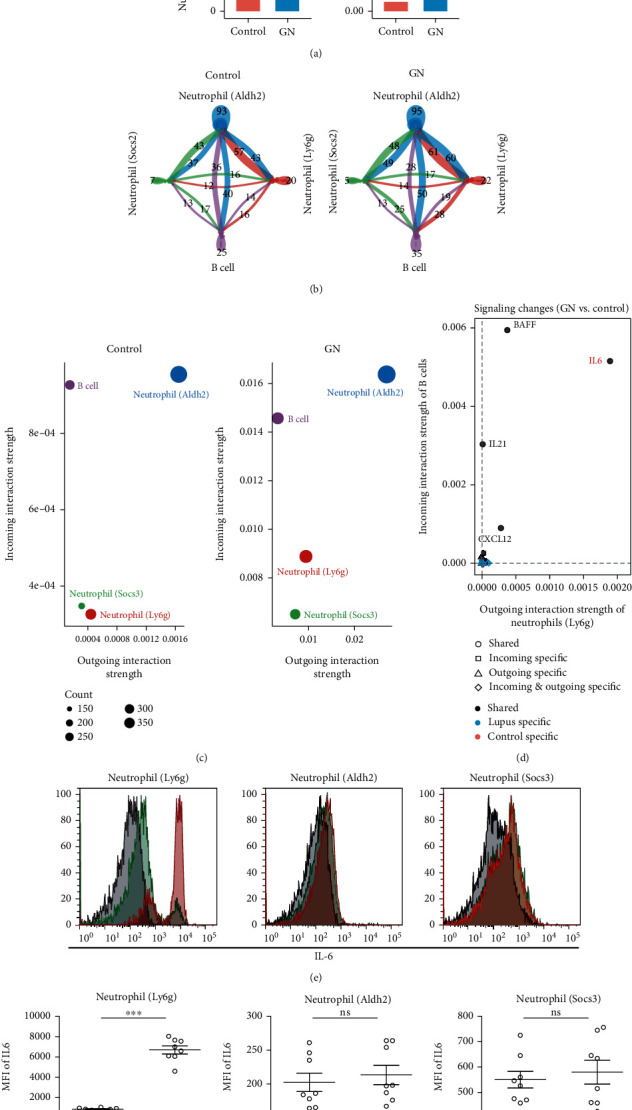
Neutrophil-derived IL-6 conducted a major signal between neutrophils and B cells in lupus kidney. (a) Bar plots showed the global communications (interaction numbers and strength) between neutrophils and B cells in control kidney and in lupus kidney. (b) Circular plots showed inferred signal numbers among neutrophil (Ly6g), neutrophil (Aldh2), neutrophil (Socs2), and B cells, in control and lupus kidney, respectively. (c) Dot plots showed outgoing and incoming inferred interaction strength among neutrophil (Ly6g), neutrophil (Aldh2), neutrophil(Socs2), and B cells, in control and lupus kidneys, respectively. (d) Dot plot showed neutrophil- (Ly6g) derived IL-6 was the major signal targeting on B cells. (e) Flow cytometry analysis validated neutrophil (Ly6g) was the major source of IL-6 in lupus kidney. Dead cells were removed by counterstaining fixable viability dye. (f) Dot plots showed the statistical analysis of resource of upregulated IL-6 from 3 neutrophil subclusters in lupus kidney (*n* = 8), compared with control kidney (*n* = 8). Data represents similar results from at least 3 independent experiments. ^∗∗∗^*p* < 0.001; ns: no significance.

Isolated neutrophil subclusters and B cells were further analyzed with the R package CellChat [17] to quantitatively explore cell-cell communications. As expected, the cell-cell interaction numbers between neutrophils and B cells were drastically increased, and the interaction strength was upregulated (Figures 2(a) and 2(b)). The increased signals were mainly distributed between neutrophil (Ly6g) and B cells (Figure 2(b)). In lupus kidney, the signals derived from neutrophil (Ly6g) were significantly increased, while those derived from neutrophil (Socs3) were decreased, and the signals derived from neutrophil (Aldh2) seem similar to healthy controls (Figure 2(c)). Then, the signals between neutrophil (Ly6g) and B cells were examined and compared. Among which, IL-6 derived from neutrophil (Ly6g) “rise” to be the major signal interacting with B cells in the lupus kidney (Figure 2(d)). Consistently, the genetic expression pattern showed IL-6 was mainly produced by neutrophil (Ly6g), and B cell significantly express the receptor for IL-6 (Supplementary figure 3) in GN. Furthermore, flow cytometry analysis of the lupus kidney showed the main source of IL-6 was expressed by neutrophil (Ly6g) (Figure 2(e)), and the statistical analysis showed that neutrophil (Ly6g) should be the main “contributor” for providing IL-6, rather than the other 2 subpopulations, in the lupus kidney (Figures 2(e) and 2(f)).

### 3.3. Neutrophil-Derived IL-6 Induces Ferroptosis Resistance in Lupus Kidney B Cells

**Figure 3:**
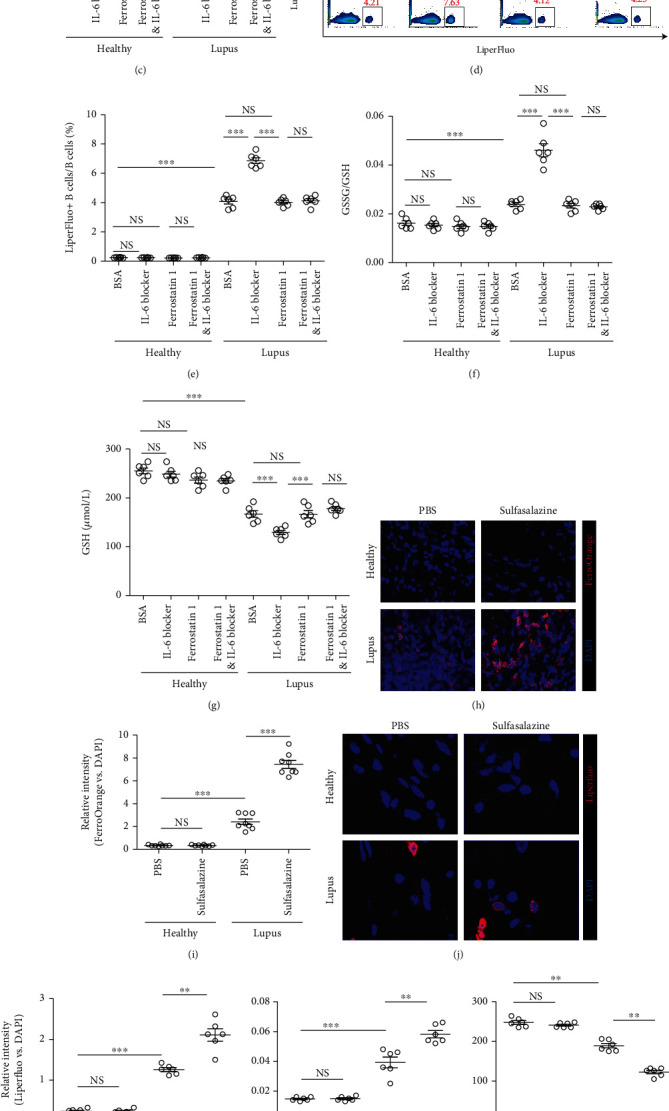
Neutrophil-derived IL-6 induces ferroptosis resistance in lupus kidney B cells. (a) The correlation matrix from scRNA-seq data showed IL-6 level correlated to the expression level of IL6R, STAT3, and SLC7A11 (xCT). (b) Flow cytometry analysis showed blocking IL-6 enhanced ferrous ions (FerroOrange) in lupus kidney B cells, which could be rescued by ferrostatin 1 treatment. Neutrophil and B cells were cocultured in transwell plates, in which B cells were placed in the bottom. (c) Dot plot showed statistical analysis of frequency of FerroOrange+ B cells from coculturing B cells and neutrophils, with or without IL-6 blocking. Each dot represents one readout. Data represents similar results from at least 3 independent experiments. ^∗∗∗^*p* < 0.001; NS: no significance. (d) Flow cytometry analysis showed blocking IL-6 enhanced lipid peroxidation (LiperFluo) in lupus kidney B cells, which could be rescued by ferrostatin 1 treatment neutrophil and B cells were cocultured in transwell plates, in which B cells were placed in the bottom. (e) Dot plot showed statistical analysis of frequency of LiperFluo+ B cells from coculturing B cells and neutrophils, with or without IL-6 blocking. Each dot represents one readout. Data represents similar results from at least 3 independent experiments. ^∗∗∗^*p* < 0.001; NS: no significance. (f) Dot plot showed statistical analysis of intracellular GSSG/GSH ratio from cocultured B cells and neutrophils, with or without IL-6 blocking. Each dot represents one readout. Data represents similar results from at least 3 independent experiments. ^∗∗∗^*p* < 0.001; NS: no significance. (g) Dot plot showed statistical analysis of intracellular GSH from cocultured B cells and neutrophils, with or without IL-6 blocking. Each dot represents one readout. Data represents similar results from at least 3 independent experiments. ^∗∗∗^*p* < 0.001; NS: no significance. (h) Immunofluorescent imaging analysis showed sulfasalazine (xCT inhibitor) treatment enhanced ferrous ions (FerroOrange) in lupus kidney B cells. Neutrophil and B cells were cocultured in transwell plates, in which B cells were placed in the bottom. Dead cells were removed by counterstaining fixable viability dye. (i) Dot plot showed statistical analysis of the relative fluorescent intensity of FerroOrange from coculturing B cells and neutrophils, with or without sulfasalazine treatment. Each dot represents one readout. Data represents similar results from at least 3 independent experiments. ^∗∗∗^*p* < 0.001; NS: no significance. (j) Immunofluorescent imaging analysis showed sulfasalazine (xCT inhibitor) treatment enhanced lipid peroxidation (LiperFluo) in lupus kidney B cells. Neutrophil and B cells were cocultured in transwell plates, in which B cells were placed in the bottom. Dead cells were removed by counterstaining fixable viability dye. (k) Dot plot showed statistical analysis of the relative fluorescent intensity of LiperFluo from coculturing B cells and neutrophils, with or without sulfasalazine treatment. Each dot represents one readout. Data represents similar results from at least 3 independent experiments. ^∗∗^*p* < 0.01; ^∗∗∗^*p* < 0.001; NS: no significance. (l) Dot plot showed statistical analysis of intracellular GSSG/GSH ratio from cocultured B cells and neutrophils, with or without sulfasalazine treatment. Each dot represents one readout. Data represents similar results from at least 3 independent experiments. ^∗∗^*p* < 0.01; ^∗∗∗^*p* < 0.001; NS: no significance. (m) Dot plot showed statistical analysis of intracellular GSH ratio from cocultured B cells and neutrophils, with or without sulfasalazine treatment. Each dot represents one readout. Data represents similar results from at least 3 independent experiments. ^∗∗^*p* < 0.01; ^∗∗∗^*p* < 0.001; NS: no significance. (n) Flow cytometry analysis showed that lupus kidney B cell proliferation was inhibited after sulfasalazine treatment. Dead cells were removed by counterstaining fixable viability dye. (o) Dot plot showed statistical analysis of the proliferated B cell from coculturing B cells and neutrophils, with or without sulfasalazine treatment. 8 wells were in each group. Data represents similar results from at least 3 independent experiments. ^∗∗∗^*p* < 0.001; NS: no significance.

To explore the inferred pathways in B cells that are inducted by neutrophil-derived IL-6, we performed a correlation test between IL-6 and the altered gene expression level in B cells. Strikingly, the B cell-derived IL-6R, STAT3, and SLC7A11 positively correlated to neutrophil-derived IL-6 (Figure 3(a)). It has been proven that the IL-6/STAT3/SLC7A11 pathway could conduct ferroptosis resistance [16, 18, 19].

In order to examine the potential role of neutrophil-derived IL-6 in regulation of suppressing ferroptosis in lupus B cells, we performed coculture of B cells and neutrophil isolated from both healthy and lupus kidney. As expected, blocking IL-6 significantly increased the Fe^2+^ signals (Figures 3(b) and 3(c)) and lipid peroxidation (Figures 3(d) and 3(e)) in B cells isolated from lupus animal, together with altered GSH and GSSG/GSH ratio (Figures 3(f) and 3(g)), key indicator for ferroptosis [20, 21] but had no obvious effect on B cells isolated from healthy controls, suggesting IL-6 could suppress ferroptosis/ferroptosis resistance in lupus B cells.

To confirm this hypothesis, ferrostatin 1, an inhibitor of erastin-induced ferroptosis [22], was added into coculture experiments, and the above phenomena were significantly rescued (Figures 3(b)–3(g)), proving that neutrophil-derived IL-6 could mediate ferroptosis resistance in lupus kidney B cells. Besides, culturing B cells either with purified neutrophils or IL-6 alone showed similar effect on the expression level of Fe^2+^ (Supplementary figures 4A and 4B), lipid peroxidation (Supplementary figures 4C and 4D), intracellular GSSG/GSH ratio (Supplementary figure 4E), and intracellular GSH (Supplementary figure 4F) in B cells, indicating IL-6 should be the “main player” derived from neutrophils mediating ferroptosis resistance.

Considering B cells in the lupus kidney exhibited higher expression of SLC7A11 (Supplementary figures 5A and 5B), a downstreaming molecule mediated by the IL-6 in mediating ferroptosis resistance [16]. We treated the cocultured B cells and neutrophils with sulfasalazine (an inhibitor of SLC7A11 [21, 23]). After sulfasalazine, the expression level of SLC7A11 was significantly downregulated (Supplementary figure 6), the Fe^2+^ signal (Figures 3(h) and 3(i)) and lipid peroxidation (Figures 3(j) and 3(k)) was significantly increased in B cells isolated from lupus mouse, meanwhile the intracellular GSSH and GSH were also altered accordingly (Figures 3(l) and 3(m)), indicating IL-6 facilitates ferroptosis resistance in lupus B cells via SLC7A11. Furthermore, the proliferation/mitosis of B cell were examined. Flow cytometry analysis showed B cell proliferation/mitosis was significantly decreased by blocking SLC7A11 (Figures 3(n) and 3(o)).

### 3.4. Inhibition of SLC7A11 Enhanced Ferroptosis of Kidney B Cells in Lupus Mice

**Figure 4:**
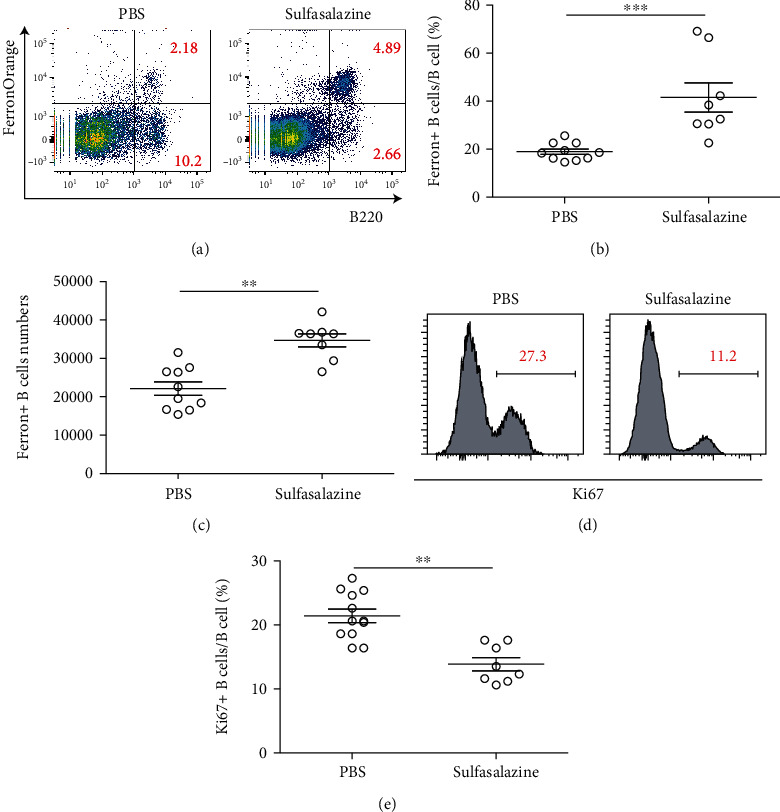
Inhibition of SLC7A11 enhanced ferroptosis of kidney B cells in lupus mice. (a) Flow cytometry analysis showed Sulfasalazine treatment (inhibiting xCT) upregulated B cell ferroptosis in lupus mice. Dead cells were removed by counterstaining fixable viability dye. (b) Dot plot showed statistical analysis of the ratio of FerronOrange+ B cell/total B cells in lupus kidney, either treated with PBS (*n* = 10), or with sulfasalazine (*n* = 8). Data represents similar results from at least 2 independent experiments. ^∗∗∗^*p* < 0.001. (c) Dot plot showed statistical analysis of the absolute cell numbers of FerronOrange+ B cell in lupus kidney, either treated with PBS (*n* = 10), or with sulfasalazine (*n* = 8). Data represents similar results from at least 2 independent experiments. ^∗∗^*p* < 0.01. (d) Flow cytometry analysis showed inhibited B cell proliferation in lupus kidney, after sulfasalazine treatment. Dead cells were removed by counterstaining fixable viability dye. (e) Dot plots showed statistical analysis of the proliferating B cells frequencies(Ki67+ B cells) and numbers in lupus kidney, either treated with PBS (*n* = 10), or with sulfasalazine (*n* = 8). Data represents similar results from at least 2 independent experiments. ^∗∗^*p* < 0.01.

In order to examine whether IL-6 could conduct ferroptosis resistance in lupus kidney B cells via SLC7A11 *in vivo*, we treated lupus animals with sulfasalazine and found the Fe^2+^-enriched B cell numbers were drastically increased after blocking SLC7A11 (Figures 4(a)–4(c)), indicating SLC7A11 could be the key factor that drives B cells to develop ferroptosis, induced by IL-6, the cytokine mainly expressed by neutrophil (Ly6g). Moreover, B cell proliferation/mitosis was also measured by counterstaining Ki67, an antibody for measuring cell proliferation/mitosis [23]. Flow cytometry analysis exhibited that after blocking SLC7A11, the B cells proliferation/mitosis was significantly decreased (Figures 4(d) and 4(e)), indicating that blocking SLC7A11 could be a potential method to suppress B cells in lupus kidney.

## 4. Discussion

Both ferroptosis and ferroptosis resistance have been observed in studies on renal diseases. Muller et al. confirmed the existence of ferroptosis in acute kidney injury [24]; the study from Schreiber et al. was consistent with that ferroptosis could contribute to the development of polycystic kidney diseases [25, 26]; and Miess et al. reported ferroptosis resistance in clear cell renal cell carcinoma [26, 27]. However, the ferroptosis resistance in lupus is still less discussed. This study helps narrow the gap in understanding the role of ferroptosis/ferroptosis resistance in lupus.

Ferroptosis resistance has been shown to be involved in the pathogenesis of various diseases. A study by Huang et al. investigated the role of UBIAD1, an enzyme that was previously identified as an apoptosis mediator; however, it could significantly alleviate ferroptosis cerebral reperfusion insult [28]. Another study by Wang et al. showed that Wnt/beta-catenin signaling, usually considered proinflammatory, could facilitate ferroptosis resistance by targeting GPX4 in gastric cancer [29]. These studies proposed that both apoptotic factors and proinflammatory factors could exert ferroptosis resistance.

IL-6, known as a proinflammatory factor, has been proven to participate in the regulation of ferroptosis [30]. It has been reported that IL-6 could promote ferroptosis in goat mammary epithelial cells via NRF2 signaling [31] and promote bronchial epithelial cells by inducing ROS-dependent lipid peroxidation [32]. On the other hand, Li et al. reported that IL-6 could exert ferroptosis resistance in head and neck squamous cell carcinoma via the IL-6/STAT3/SLC7A11 axis [16]. This study identified that IL-6 could protect B cells from ferroptosis in lupus kidney via the IL-6/STAT3/SLC7A11 axis. Taken together, these studies highlighted the complex role of IL-6 in regulating ferroptosis and suggested that the effect of IL-6 on ferroptosis may be context-dependent.

Previous studies have reported neutrophils could modulate B cells [33, 34] by providing BAFF, APRIL, which help activate B cells and even drive the antibody class switch and generation of IgG2 and IgA [34]. This study, however, discovered that a population of neutrophil could provide IL-6 for B cells and suppress ferroptosis in lupus kidney, via SLC7A11. This discovery would improve the understanding of the cross-talk between neutrophils and B cells and help provide an alternative strategy for suppressing the pathological features of B cells in lupus [35, 36].

The scRNA-seq data analyzed in this study was obtained from surgically isolated murine glomeruli from murine in the development of GN induced by injury, not from the whole kidney, and GN is different from lupus; however, the downstreaming cell-cell communication analysis between neutrophils and B cells and the coculture experiment showed consistent result that neutrophil-derived IL-6 could promote ferroptosis resistance in lupus kidney B cells, suggesting this could possibly be a general phenomenon in the development of glomerular injury. Besides, the morphological feature of mitochondria should be further tested to solidify the status of ferroptosis, and genetic silencing/knockout of SLC7A11 in B cells should be performed and provided to rule out off-target effects independent of SLC7A11.

This study indicated that IL-6 derived from neutrophils could locally modulate the ferroptosis resistance in B cells in the lupus kidney, mainly via SLC7A11. This research shed lights in delineating the microenvironment around B cells and provided a candidate solution for improving therapeutics against lupus.

## Data Availability

The datasets and code generated or analysed in this study are available from the corresponding author upon reasonable request.

## Supplementary Materials

Supplementary Materials Supplementary Table: cell numbers and genes identified from scRAN-seq analysis. The cell numbers and gene numbers were counted/identified based on integration of control and lupus kidney scRNA-seq datasets. Supplementary Figure 1: quality control over scRNA-seq data. (A) Violin plots showed the sequencing quality of control mouse. (B) Scatter plot showed the relationships between counts and genes sequenced from control mouse. (C) Violin plots showed the sequencing quality of lupus mouse. (D) Scatter plot showed the relationships between counts and genes sequenced from lupus mouse. Supplementary Figure 2: inferred cell-cell communications among all the cell types identified from scRNA-seq data. A. Circular plots showed the differences of inferred cell-cell interaction numbers and strength across all the cell types (lupus vs. control). The red color indicates upregulated signals, and the blue color indicates downregulated signals. (B) Bar plots showed the global communications (interaction numbers and strength) across all the cell types (lupus vs. control). Supplementary Figure 3: the expression of IL-6 and IL-6 receptor. Feature plots showed the expression pattern of IL-6 and IL-6 receptor across all cells identified from scRNA-seq data. Supplementary Figure 4: culturing B cell with IL-6 exerted similar effect as coculture with neutrophils. (A) Flow cytometry analysis showed ferrous ions (FerroOrange) signals in B cells from either cultured with IL-6 alone or cocultured with neutrophils. (B) Dot plot showed statistical analysis of FerroOrange+ B cells from either cultured with IL-6 alone or cocultured with neutrophils. Each dot represents one readout. Data represents similar results from at least 3 independent experiments. ^∗∗∗^*p* < 0.001; NS: no significance. (C) Flow cytometry analysis showed lipid peroxidation (LiperFluo) in B cells from either cultured with IL-6 alone or cocultured with neutrophils. (D) Dot plot showed statistical analysis of LiperFluo+ B cells from either cultured with IL-6 alone or cocultured with neutrophils. Each dot represents one readout. Data represents similar results from at least 3 independent experiments. ^∗∗∗^*p* < 0.001; NS: no significance. (E) Dot plot showed statistical analysis of intracellular GSSG/GSH ratio from B cells either cultured with IL-6 alone or cocultured with neutrophils. Each dot represents one readout. Data represents similar results from at least 3 independent experiments. ^∗∗^*p* < 0.01; NS: no significance. (F) Dot plot showed statistical analysis of intracellular GSH from B cells either cultured with IL-6 alone or cocultured with neutrophils. Each dot represents one readout. Data represents similar results from at least 3 independent experiments. ^∗∗^*p* < 0.01; NS: no significance. Supplementary Figure 5: B cells express higher SLC7A11 in the lupus kidney. (A) Flow cytometry analysis of the expression of SLC7A11 in kidney. (B) Statistical analysis of expression of SLC7A11 in kidney B cells. 5 mice in each group; ^∗∗∗^*p* < 0.001, data represents similar results from 2 independent experiments. Supplementary Figure 6: sulfasalazine inhibited the expression of SLC7A11 from B cells. qPCR measures showed the expression level of SLC7A11 from B cells was significantly inhibited by treatment of sulfasalazine.
