# Supplementary figures and images for: Neutrophil-Derived IL-6 Potentially Drives Ferroptosis Resistance in B Cells in Lupus Kidney
Source: Mediators Inflamm. 2023 May 27;2023:9810733. doi: 10.1155/2023/9810733 (PMC10239302; doi:10.1155/2023/9810733)

Supplementary figure 1

A

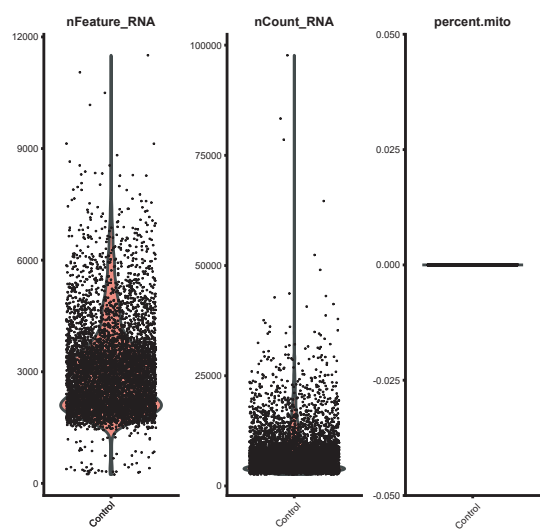

B

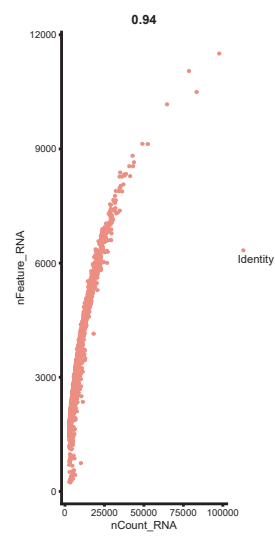

C

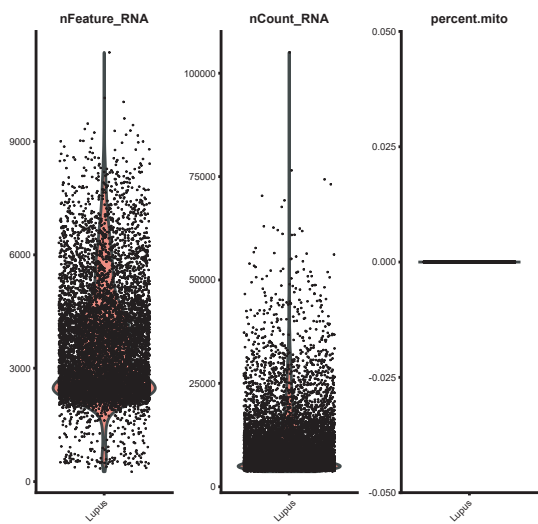

D

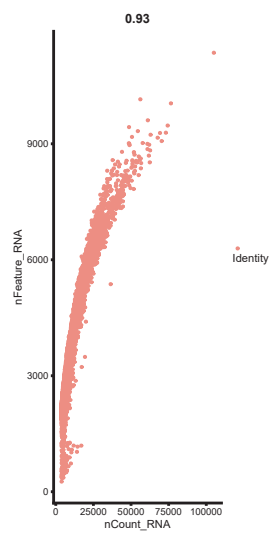

Supplement: Supplementary Materials — Supplementary Table: cell numbers and genes identified from scRAN-seq analysis. The cell numbers and gene numbers were counted/identified based on integration of control and lupus kidney scRNA-seq datasets. Supplementary Figure 1: quality control over scRNA-seq data. (A) Violin plots showed the sequencing quality of control mouse. (B) Scatter plot showed the relationships between counts and genes sequenced from control mouse. (C) Violin plots showed the sequencing quality of lupus mouse. (D) Scatter plot showed the relationships between counts and genes sequenced from lupus mouse. Supplementary Figure 2: inferred cell-cell communications among all the cell types identified from scRNA-seq data. A. Circular plots showed the differences of inferred cell-cell interaction numbers and strength across all the cell types (lupus vs. control). The red color indicates upregulated signals, and the blue color indicates downregulated signals. (B) Bar plots showed the global communications (interaction numbers and strength) across all the cell types (lupus vs. control). Supplementary Figure 3: the expression of IL-6 and IL-6 receptor. Feature plots showed the expression pattern of IL-6 and IL-6 receptor across all cells identified from scRNA-seq data. Supplementary Figure 4: culturing B cell with IL-6 exerted similar effect as coculture with neutrophils. (A) Flow cytometry analysis showed ferrous ions (FerroOrange) signals in B cells from either cultured with IL-6 alone or cocultured with neutrophils. (B) Dot plot showed statistical analysis of FerroOrange+ B cells from either cultured with IL-6 alone or cocultured with neutrophils. Each dot represents one readout. Data represents similar results from at least 3 independent experiments. ∗∗∗p < 0.001; NS: no significance. (C) Flow cytometry analysis showed lipid peroxidation (LiperFluo) in B cells from either cultured with IL-6 alone or cocultured with neutrophils. (D) Dot plot showed statistical analysis of LiperFluo+ B cells from [file 9810733.f1.zip › Supplementary Figure 1 - Quality Control over scRAN-seq data.pdf]

### Supplementary figure 2

A

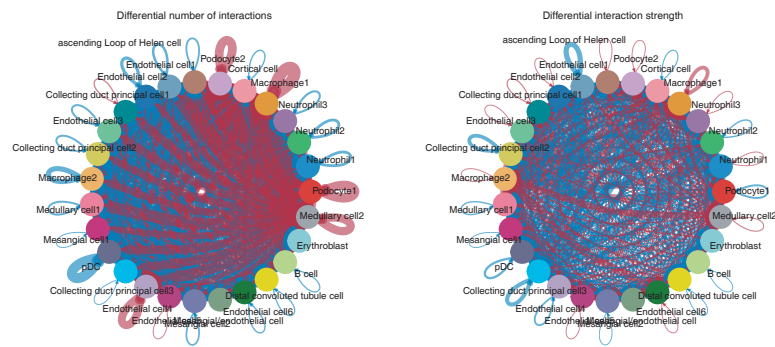

B

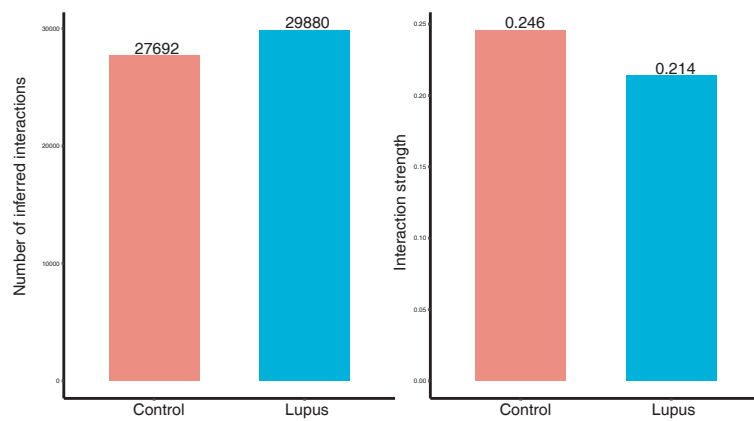

Supplement: Supplementary Materials — Supplementary Table: cell numbers and genes identified from scRAN-seq analysis. The cell numbers and gene numbers were counted/identified based on integration of control and lupus kidney scRNA-seq datasets. Supplementary Figure 1: quality control over scRNA-seq data. (A) Violin plots showed the sequencing quality of control mouse. (B) Scatter plot showed the relationships between counts and genes sequenced from control mouse. (C) Violin plots showed the sequencing quality of lupus mouse. (D) Scatter plot showed the relationships between counts and genes sequenced from lupus mouse. Supplementary Figure 2: inferred cell-cell communications among all the cell types identified from scRNA-seq data. A. Circular plots showed the differences of inferred cell-cell interaction numbers and strength across all the cell types (lupus vs. control). The red color indicates upregulated signals, and the blue color indicates downregulated signals. (B) Bar plots showed the global communications (interaction numbers and strength) across all the cell types (lupus vs. control). Supplementary Figure 3: the expression of IL-6 and IL-6 receptor. Feature plots showed the expression pattern of IL-6 and IL-6 receptor across all cells identified from scRNA-seq data. Supplementary Figure 4: culturing B cell with IL-6 exerted similar effect as coculture with neutrophils. (A) Flow cytometry analysis showed ferrous ions (FerroOrange) signals in B cells from either cultured with IL-6 alone or cocultured with neutrophils. (B) Dot plot showed statistical analysis of FerroOrange+ B cells from either cultured with IL-6 alone or cocultured with neutrophils. Each dot represents one readout. Data represents similar results from at least 3 independent experiments. ∗∗∗p < 0.001; NS: no significance. (C) Flow cytometry analysis showed lipid peroxidation (LiperFluo) in B cells from either cultured with IL-6 alone or cocultured with neutrophils. (D) Dot plot showed statistical analysis of LiperFluo+ B cells from [file 9810733.f1.zip › Supplementary Figure 2 - Cell-cell communications among all cell types.pdf]

Supplementary figure 3

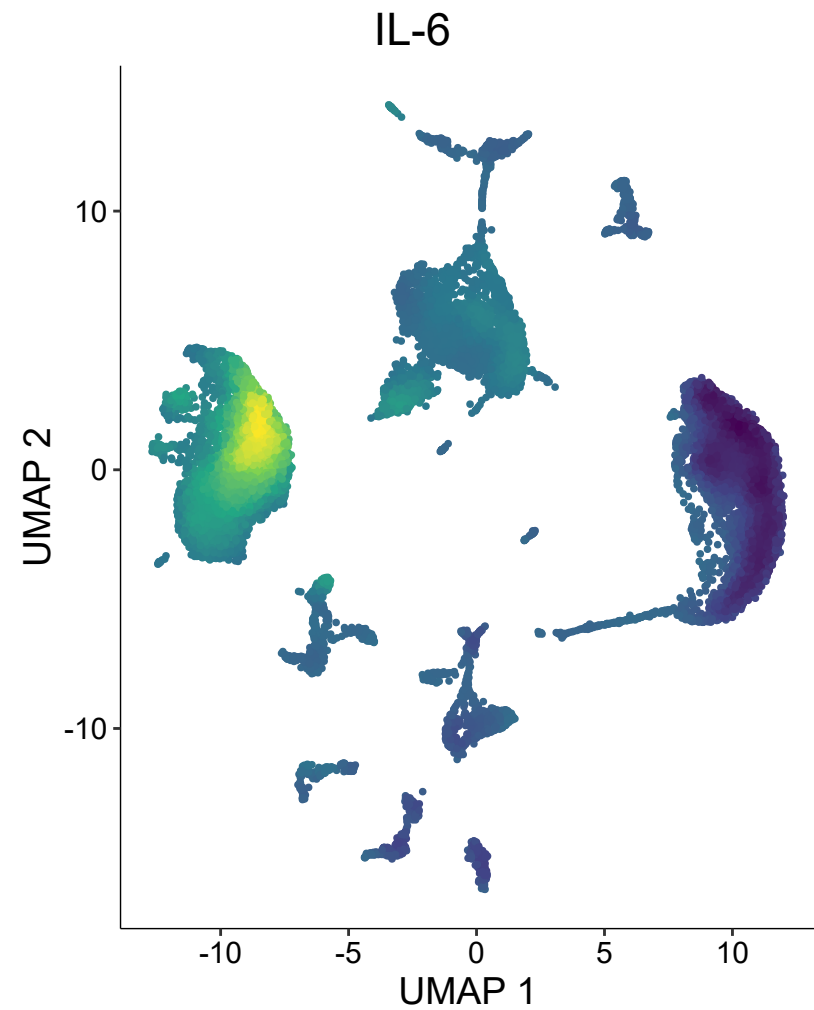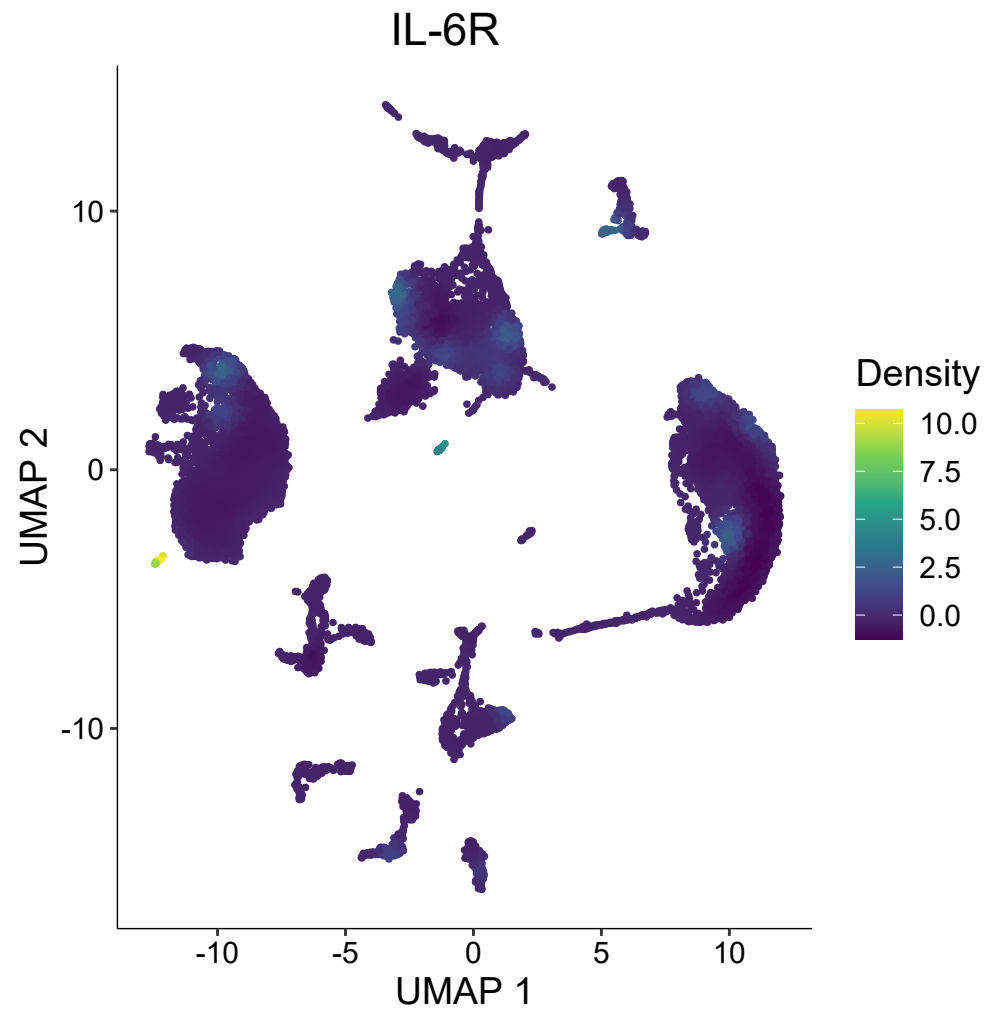

Supplement: Supplementary Materials — Supplementary Table: cell numbers and genes identified from scRAN-seq analysis. The cell numbers and gene numbers were counted/identified based on integration of control and lupus kidney scRNA-seq datasets. Supplementary Figure 1: quality control over scRNA-seq data. (A) Violin plots showed the sequencing quality of control mouse. (B) Scatter plot showed the relationships between counts and genes sequenced from control mouse. (C) Violin plots showed the sequencing quality of lupus mouse. (D) Scatter plot showed the relationships between counts and genes sequenced from lupus mouse. Supplementary Figure 2: inferred cell-cell communications among all the cell types identified from scRNA-seq data. A. Circular plots showed the differences of inferred cell-cell interaction numbers and strength across all the cell types (lupus vs. control). The red color indicates upregulated signals, and the blue color indicates downregulated signals. (B) Bar plots showed the global communications (interaction numbers and strength) across all the cell types (lupus vs. control). Supplementary Figure 3: the expression of IL-6 and IL-6 receptor. Feature plots showed the expression pattern of IL-6 and IL-6 receptor across all cells identified from scRNA-seq data. Supplementary Figure 4: culturing B cell with IL-6 exerted similar effect as coculture with neutrophils. (A) Flow cytometry analysis showed ferrous ions (FerroOrange) signals in B cells from either cultured with IL-6 alone or cocultured with neutrophils. (B) Dot plot showed statistical analysis of FerroOrange+ B cells from either cultured with IL-6 alone or cocultured with neutrophils. Each dot represents one readout. Data represents similar results from at least 3 independent experiments. ∗∗∗p < 0.001; NS: no significance. (C) Flow cytometry analysis showed lipid peroxidation (LiperFluo) in B cells from either cultured with IL-6 alone or cocultured with neutrophils. (D) Dot plot showed statistical analysis of LiperFluo+ B cells from [file 9810733.f1.zip › Supplementary Figure 3 - Expression of IL-6 and IL-6 Receptor.pdf]

Supplementary figure 4

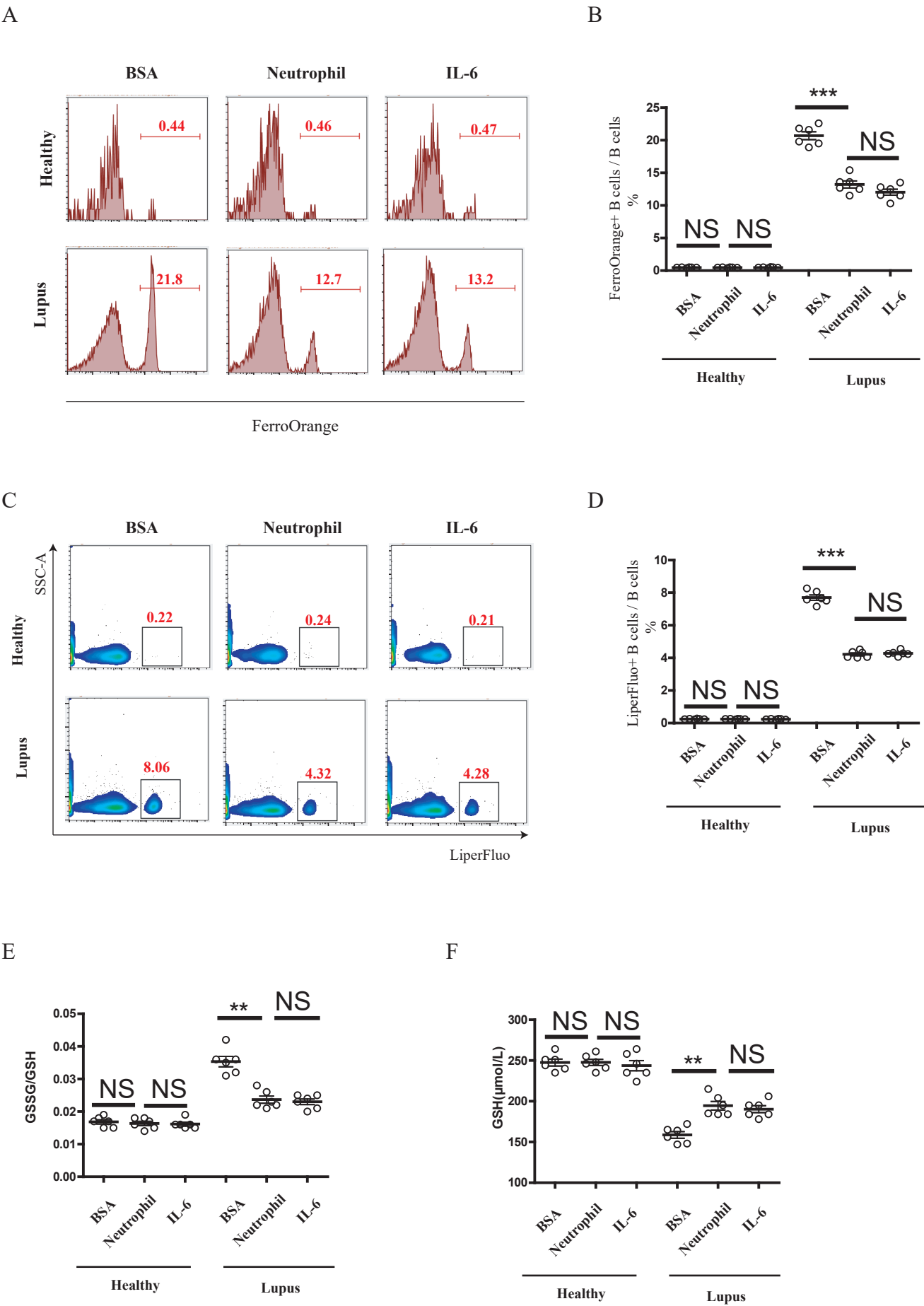

Supplement: Supplementary Materials — Supplementary Table: cell numbers and genes identified from scRAN-seq analysis. The cell numbers and gene numbers were counted/identified based on integration of control and lupus kidney scRNA-seq datasets. Supplementary Figure 1: quality control over scRNA-seq data. (A) Violin plots showed the sequencing quality of control mouse. (B) Scatter plot showed the relationships between counts and genes sequenced from control mouse. (C) Violin plots showed the sequencing quality of lupus mouse. (D) Scatter plot showed the relationships between counts and genes sequenced from lupus mouse. Supplementary Figure 2: inferred cell-cell communications among all the cell types identified from scRNA-seq data. A. Circular plots showed the differences of inferred cell-cell interaction numbers and strength across all the cell types (lupus vs. control). The red color indicates upregulated signals, and the blue color indicates downregulated signals. (B) Bar plots showed the global communications (interaction numbers and strength) across all the cell types (lupus vs. control). Supplementary Figure 3: the expression of IL-6 and IL-6 receptor. Feature plots showed the expression pattern of IL-6 and IL-6 receptor across all cells identified from scRNA-seq data. Supplementary Figure 4: culturing B cell with IL-6 exerted similar effect as coculture with neutrophils. (A) Flow cytometry analysis showed ferrous ions (FerroOrange) signals in B cells from either cultured with IL-6 alone or cocultured with neutrophils. (B) Dot plot showed statistical analysis of FerroOrange+ B cells from either cultured with IL-6 alone or cocultured with neutrophils. Each dot represents one readout. Data represents similar results from at least 3 independent experiments. ∗∗∗p < 0.001; NS: no significance. (C) Flow cytometry analysis showed lipid peroxidation (LiperFluo) in B cells from either cultured with IL-6 alone or cocultured with neutrophils. (D) Dot plot showed statistical analysis of LiperFluo+ B cells from [file 9810733.f1.zip › Supplementary Figure 4 - IL-6 and coculture.pdf]

Supplementary figure 5

A

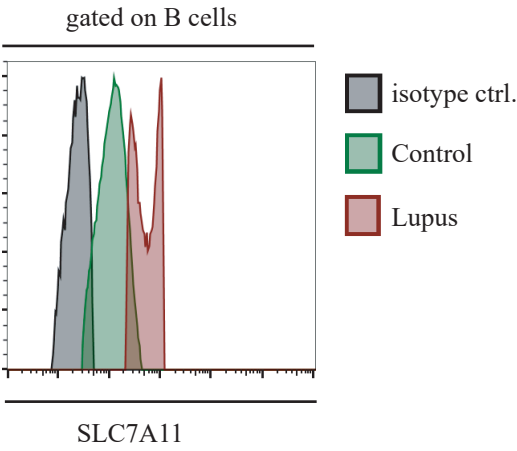

B

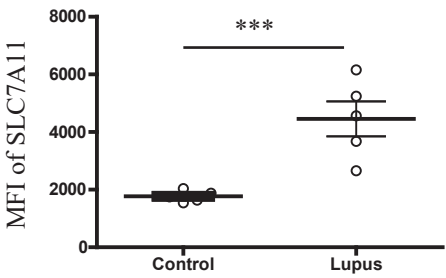

Supplement: Supplementary Materials — Supplementary Table: cell numbers and genes identified from scRAN-seq analysis. The cell numbers and gene numbers were counted/identified based on integration of control and lupus kidney scRNA-seq datasets. Supplementary Figure 1: quality control over scRNA-seq data. (A) Violin plots showed the sequencing quality of control mouse. (B) Scatter plot showed the relationships between counts and genes sequenced from control mouse. (C) Violin plots showed the sequencing quality of lupus mouse. (D) Scatter plot showed the relationships between counts and genes sequenced from lupus mouse. Supplementary Figure 2: inferred cell-cell communications among all the cell types identified from scRNA-seq data. A. Circular plots showed the differences of inferred cell-cell interaction numbers and strength across all the cell types (lupus vs. control). The red color indicates upregulated signals, and the blue color indicates downregulated signals. (B) Bar plots showed the global communications (interaction numbers and strength) across all the cell types (lupus vs. control). Supplementary Figure 3: the expression of IL-6 and IL-6 receptor. Feature plots showed the expression pattern of IL-6 and IL-6 receptor across all cells identified from scRNA-seq data. Supplementary Figure 4: culturing B cell with IL-6 exerted similar effect as coculture with neutrophils. (A) Flow cytometry analysis showed ferrous ions (FerroOrange) signals in B cells from either cultured with IL-6 alone or cocultured with neutrophils. (B) Dot plot showed statistical analysis of FerroOrange+ B cells from either cultured with IL-6 alone or cocultured with neutrophils. Each dot represents one readout. Data represents similar results from at least 3 independent experiments. ∗∗∗p < 0.001; NS: no significance. (C) Flow cytometry analysis showed lipid peroxidation (LiperFluo) in B cells from either cultured with IL-6 alone or cocultured with neutrophils. (D) Dot plot showed statistical analysis of LiperFluo+ B cells from [file 9810733.f1.zip › Supplementary Figure 5 - B cells express higher SLC7A11 in lupus kidney.pdf]

**Supplementary figure 6**

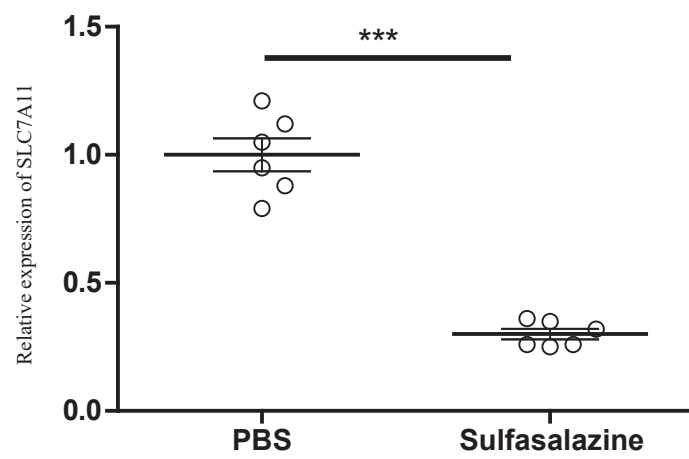

Supplement: Supplementary Materials — Supplementary Table: cell numbers and genes identified from scRAN-seq analysis. The cell numbers and gene numbers were counted/identified based on integration of control and lupus kidney scRNA-seq datasets. Supplementary Figure 1: quality control over scRNA-seq data. (A) Violin plots showed the sequencing quality of control mouse. (B) Scatter plot showed the relationships between counts and genes sequenced from control mouse. (C) Violin plots showed the sequencing quality of lupus mouse. (D) Scatter plot showed the relationships between counts and genes sequenced from lupus mouse. Supplementary Figure 2: inferred cell-cell communications among all the cell types identified from scRNA-seq data. A. Circular plots showed the differences of inferred cell-cell interaction numbers and strength across all the cell types (lupus vs. control). The red color indicates upregulated signals, and the blue color indicates downregulated signals. (B) Bar plots showed the global communications (interaction numbers and strength) across all the cell types (lupus vs. control). Supplementary Figure 3: the expression of IL-6 and IL-6 receptor. Feature plots showed the expression pattern of IL-6 and IL-6 receptor across all cells identified from scRNA-seq data. Supplementary Figure 4: culturing B cell with IL-6 exerted similar effect as coculture with neutrophils. (A) Flow cytometry analysis showed ferrous ions (FerroOrange) signals in B cells from either cultured with IL-6 alone or cocultured with neutrophils. (B) Dot plot showed statistical analysis of FerroOrange+ B cells from either cultured with IL-6 alone or cocultured with neutrophils. Each dot represents one readout. Data represents similar results from at least 3 independent experiments. ∗∗∗p < 0.001; NS: no significance. (C) Flow cytometry analysis showed lipid peroxidation (LiperFluo) in B cells from either cultured with IL-6 alone or cocultured with neutrophils. (D) Dot plot showed statistical analysis of LiperFluo+ B cells from [file 9810733.f1.zip › Supplementary Figure 6 - Sulfasalazine inhibits SLC7A11.pdf]
